# Perceptions related to health, illness, and provision of healthcare among West African migrants residing in Norway: a qualitative study

**DOI:** 10.1186/s12913-025-13329-w

**Published:** 2025-10-09

**Authors:** Emmanuel Aoudi Chance, Lise-Merete Alpers, Abdallah Abudayya, Tesfaye Hordofa Leta, Dia Florence, Siri Nyen, Zada Pajalic

**Affiliations:** 1 Faculty of Health Sciences, VID Specialized University, Ulriksdal 10, Bergen,, 5009 Norway; 2 VID Specialized University, Thurmannsgate 12 A, Oslo, Oslo, Norway; 3 Université de Bertoua, Postboks 652, Bertoua, Cameroon; 4 Norwegian Mission Society, Postboks 226 Sentrum, Stavanger, 4001 Norway; 5 Universitetet i Sørøst-Norge, Postboks 4, Borre, 3199 Norway

**Keywords:** Immigrant, Illness behavior, Healthcare experience, Cultural competency, Cultural sensitivity

## Abstract

**Background:**

Norway has seen an increase in its West African immigrant population, whose integration into the community is influenced by their beliefs about health, illness, and healthcare. This study aimed to explore how West African immigrants experience and navigate healthcare in Norway.

**Methods:**

This qualitative study involved semi-structured interviews with 31 West African immigrants residing in Norway. Thematic analysis was employed to examine the broader context of their resettlement experiences, health perceptions, health-seeking behaviors, and overall experiences with the Norwegian healthcare system.

**Results:**

Three key findings emerged: (1) Participants expressed strong cultural and religious beliefs about illness, often attributing it to spiritual causes and ancestral spirits. Many initially sought traditional remedies, viewing them as complementary to Western medicine. (2) Effective communication and trust in healthcare providers were identified as critical factors, influencing patients’ sense of being heard, respected, and involved in decision-making. (3) Participants highlighted the challenges of balancing traditional health practices with the Norwegian biomedical system. Social networks, including family, community, and religious institutions, played a pivotal role in navigating this healthcare landscape and influencing health-related decisions.

**Conclusion:**

The findings highlight the complexity of integrating traditional health beliefs with Western biomedical practices. They underscore the importance of culturally sensitive healthcare approaches that acknowledge and incorporate traditional health perspectives to enhance the healthcare experience for immigrant communities.

**Supplementary Information:**

The online version contains supplementary material available at 10.1186/s12913-025-13329-w.

## Introduction

Norway’s public healthcare system stands out for its comprehensive approach to provide health services for all residents [1, 2]. The system is designed to ensure equitable access to high-quality healthcare services for all individuals, regardless of socioeconomic status [3]. Norway’s healthcare system, built on the principle of universal access, is a beacon of fairness and equity. It is primarily funded through taxation, ensuring that all legal residents are automatically enrolled in the National Insurance Scheme (Folketryggen). This comprehensive coverage includes essential health services, such as visits to general practitioners, hospital treatment, maternity care, and certain mental health services [4, 5]. While the system ensures broad access, it is important to note that healthcare is not entirely free of charge. Adults are required to pay modest co-payments for many services until they reach an annual cap—approximately NOK 3,040 in 2023)—after which they receive an exemption card that grants free care for the remainder of the year [5].

Despite the system’s universal design, some individuals also choose to supplement their healthcare with private health insurance. This is often done to gain quicker access to specialists, private clinics, or treatments that fall outside the scope of the public scheme, such as certain dental or physiotherapy services. Employers, particularly in competitive sectors, may offer supplemental insurance as part of employee benefit packages, allowing their staff to bypass public waiting lists and receive expedited care.

However, coverage is not uniform for everyone. Residency status plays a significant role: tourists, undocumented migrants, and short-term residents typically do not qualify for public coverage. They must rely on private travel insurance or pay for care out of pocket. Additionally, personal finances and individual preferences play a significant role in healthcare choices. Those with higher incomes may opt for private insurance to access premium services or avoid waiting times. Knowledge and integration into the system also affect access; immigrants unfamiliar with how the Norwegian healthcare system works may fail to register correctly or may not fully understand their entitlements, which can result in significant gaps in coverage [6, 7].

These disparities can influence healthcare-seeking behavior in various ways. Individuals without full coverage, particularly those from low-income or immigrant backgrounds, may delay or avoid seeking care due to concerns about costs or uncertainty about their rights [7]. Cultural expectations also play a role. Some immigrants, for example, may anticipate more diagnostic tests or medications than what Norwegian doctors typically offer, which can lead to frustration or a preference for seeking care abroad [6]. However, it is encouraging to note that older adults tend to be more proactive about visiting their general practitioner, demonstrating a positive approach to health management [8].

In summary, while Norway’s healthcare system is designed to provide universal access, the experience of care can vary significantly depending on factors such as legal status, socioeconomic background, cultural background, and personal choices.

Despite having access to Norway’s biomedical healthcare system, many West African immigrants continue to use traditional medicine, comprising culturally grounded knowledge, beliefs, and practices, as a complementary approach to managing health. This reflects both the enduring relevance of pre-migration health [9] and the complex realities of navigating a new healthcare system marked by cultural and structural challenges [7]. As Næss (2019) illustrates in her study of Somali immigrants in Norway, trust is central to the process of healthcare integration but is often undermined by a pervasive mutual unfamiliarity between immigrants and healthcare providers [10].

The goal of the Norwegian government is to offer standardized healthcare services [7] and equal access to everyone. The term “immigrant” typically refers to a person who moves from one place to another, often across borders, regions, or countries, to reside or work in a new location [11, 12]. Various factors drive migration, but the specific circumstances and reasons for migration vary widely among individuals. Common reasons for migrating include the desire for better economic opportunities, the need to flee conflict or persecution, the desire to reunite with family, and the pursuit of educational and professional goals in a different location. Data from Statistics Norway [13] indicate that as of 2022, the immigrant population in Norway had reached 877,227 individuals, with 105,817 immigrants originating from countries in West Africa. The presence of immigrants may lead to significant changes in routines, competencies, attitudes, and values within Norway’s healthcare systems [13]. Understanding how West African immigrants view health and illness is crucial for their well-being and quality of life. Caring, as part of human experience, may be considered the essence of genuine love [14]; thus, it is worthwhile to explore both the scientific and humanistic nature of nursing [15].

However, perceptions of health, illness, and healthcare differ among various cultures [16]. Many West African immigrants in Norway consider both traditional healthcare and Western nursing care essential for their healthcare needs [17, 18]. Traditional healthcare, as outlined by the World Health Organization, encompasses the knowledge, skills, and practices rooted in theories, beliefs, and experiences unique to different cultures [19]. These traditions are employed to maintain good health and prevent, diagnose, enhance, or treat both physical and mental ailments, whether they can be readily explained or not [20]. Western nursing care, or biomedical care, refers to a medicinal approach rooted in the biological sciences [21]. These two forms of care (traditional and biomedical) coexist in immigrants’ attitudes and behaviors, which may sometimes lead to tension and result in inappropriate care practices and adverse health outcomes in healthcare settings, consistent with previous reports [18, 22].

Western healthcare faces distinct challenges in meeting the diverse healthcare needs of immigrants residing in Norway who seek medical care [23]. This is particularly relevant because many immigrants continue to embrace their traditional cultures, values, and lifestyles [24, 25]. When hospitalized in Western healthcare facilities, some immigrants in Norway choose traditional medicines, while others opt for Western medicines, and some may even use a combination of both [26]. Some patients with immigrant backgrounds secretly obtain medicines from relatives who understand their disease and culture [27, 28], as well as the extensive healing power of plants and animals for various health purposes [29, 30]. A meta-ethnographic review synthesizing 37 qualitative studies demonstrated that family members often serve as informal caregivers, possessing cultural and traditional knowledge that influences health decisions [31]. These relatives understand traditional culture, integrate natural health practices into their healthcare routines, and consider both body and the spirit in medical treatment; thus, many immigrants seek family support when they become ill [32, 33]. Some studies have reported how traditional knowledge on healthcare has promoted health among people in Africa [34]. In Norway, conventional biomedicine predominates in the professional healthcare system [23], and immigrants require new knowledge and information related to biomedicine as part of their introduction program [35]. Norway’s Introduction Program is a structured initiative aimed at facilitating the integration of refugees and asylum seekers into Norwegian society by providing essential skills and knowledge for effective participation in the workforce and community life [36]. Healthcare professionals must consider and acknowledge alternative understandings of illnesses and treatments. The challenge for Western or modern health providers in Norway is to incorporate or combine their practices with the traditional practices of immigrants in Norwegian hospitals and home nursing care [35].

Research has shown that healthcare professionals in Norway report a lack of cultural awareness [37, 38]. However, more knowledge is needed about the perceptions of West African immigrants regarding health, illness, and healthcare provision. By examining the cultural nuances and individual perceptions of West African immigrants, this study aims to fill a critical gap in the literature and provide valuable insights that could significantly improve the quality of healthcare services delivered to this population. This research goes beyond previous studies by employing a qualitative approach to explore the lived experiences of West African immigrants, allowing for a deeper understanding of their cultural beliefs, healthcare practices, and challenges in accessing healthcare services. Understanding and generating knowledge about the perceptions of healthcare provision and the meaning of illness or disease are highly relevant for delivering quality care to West African immigrants in Norway [23].

As of January 1, 2025, immigrants and individuals born in Norway to immigrant parents comprised 21.4% of the total population—17.3% and 4.1%, respectively [39]. This growing segment of the population is highly diverse in terms of socio-cultural, linguistic, and socioeconomic backgrounds. While healthcare systems can be challenging to navigate for all individuals—due to factors such as medical uncertainty, complex administrative procedures, and evolving clinical practices—numerous studies have shown that migrants often encounter additional, intersecting barriers. These include language difficulties, limited health literacy, unfamiliarity with healthcare structures, and experiences of discrimination [35, 40].

Importantly, these experiences are not uniform, and we acknowledge that migrant populations are not a monolithic group. However, empirical evidence suggests that structural and systemic inequities disproportionately affect migrants’ access to and use of healthcare services. These factors can shape healthcare-seeking behaviors in distinct ways that are not fully understood when examining the general population alone. This study aims to explore how West African immigrants experience and navigate healthcare in Norway.

## Methods

### Study design

This study employed a qualitative research design using Braun and Clarke’s (2006) thematic analysis to explore how West African immigrants in Norway perceive and navigate health, illness, and healthcare [41]. Thematic analysis was chosen for its flexibility and suitability in identifying, analyzing, and interpreting patterns of meaning across qualitative data. The study was guided by a constructivist paradigm, emphasizing participants’ own interpretations and experiences as shaped by their social and cultural contexts.

### Participants and recruitment

A total of 31 West African immigrants residing in Bergen, Norway, were recruited using purposive and snowball sampling. Inclusion criteria included: age 20–55, residence in Norway for a minimum of six months, and the ability to communicate in English, French, or Norwegian. Interpreter support was offered when necessary. Individuals with severe cognitive impairments or acute illness were excluded.

Recruitment took place in community settings such as churches, markets, clinics, and cultural events, with the support of community leaders and local networks. Information and invitations to participate were provided through direct personal approaches in these settings, as well as via email correspondence. Participants represented diverse socioeconomic, educational, and ethnic backgrounds from countries including Nigeria, Ghana, Senegal, Mali, The Gambia, and Sierra Leone. The final sample included both men and women, with varying lengths of residence in Norway (1–55 years), and occupations ranging from students to manual laborers and healthcare workers.

The recruitment strategy, while effective for reaching this specific community, has several potential limitations and biases. The use of purposive and snowball sampling means that the sample may not accurately represent the entire West African immigrant population in Bergen. Recruiting from community hubs, such as churches and markets, often with the assistance of community leaders, could lead to an overrepresentation of socially integrated individuals, unintentionally excluding those who are more isolated or those with differing views. Additionally, the snowball sampling method risks producing a more homogeneous sample because participants are likely to refer others from similar social networks, which can limit the diversity of experiences and perspectives gathered. Lastly, the reliance on email correspondence may introduce bias against those who are not digitally literate, potentially excluding individuals with lower socioeconomic status, recent immigrants, or older adults who are less likely to use this form of communication.

### Data collection

Data were collected through semi-structured, face-to-face interviews conducted by the first author between January and June 2023. An interview guide was developed based on a focused literature review on migrant health, healthcare access, cultural health beliefs, and help-seeking behaviors among African and other immigrant populations in Europe [42, 43]. Key considerations guiding the development of the guide included ensuring cultural sensitivity, relevance to transnational health experiences, and openness to unexpected or nuanced perspectives.

To refine the interview guide for clarity and cultural appropriateness, it was pilot-tested with two West African immigrants living in Bergen. Both individuals met the study’s inclusion criteria but were not included in the final sample. Their feedback led to adjustments in the wording, sequence, and framing of questions to improve comprehensibility and flow, and to ensure the guide captured personal and social dimensions of healthcare experience.

The final interview guide included open-ended questions designed to explore participants’ health beliefs, previous interactions with the Norwegian healthcare system, and perceived barriers and facilitators to care.

Interviews were conducted in participants’ preferred language—English, French, or Norwegian—with trained interpreters used when necessary. Settings were chosen by participants and included private homes, community centers, and public libraries. Each interview lasted approximately 30–40 min.

To build trust and encourage open dialogue, culturally sensitive techniques were employed, such as conversational tone, active listening, and adaptation to participants’ communication styles. All interviews were audio-recorded with informed consent, transcribed verbatim in their original language, and supplemented with field notes. Data saturation was considered achieved after 20 interviews, with 11 additional interviews conducted to ensure thematic depth and represent diverse perspectives.

### Data analysis

To ensure a consistent coding process, transcripts originally in French and Norwegian were professionally translated into English. A bilingual member of the research team verified the accuracy of these translations by cross-referencing the translated text with the original audio recordings, ensuring that the conceptual meaning was maintained. Following this preparation, thematic analysis was conducted using Braun and Clarke’s six-step approach [41]. An inductive, data-driven process guided coding and theme development. Transcripts were read repeatedly, and descriptive codes were generated using NVivo software. Codes were then grouped into broader themes based on recurring patterns across the dataset.

Three core themes were identified: (1) *Cultural Perceptions and Health Beliefs*, (2) *Communication Barriers and Trust in Healthcare*, and (3) *Navigating Healthcare Systems and the Role of Social Networks*. Themes were refined iteratively for internal coherence and conceptual clarity. Illustrative quotes were selected to support each theme. Analytical rigor was maintained through reflexive journaling and peer debriefing with co-authors.

### Ethical considerations

Ethical approval was granted by the Regional Committees for Medical and Health Ethics (reference No. 532019), and the study was registered with the Norwegian Agency for Shared Services in Education and Research (reference No. 925298) and VID Specialized University. Before conducting the interviews and discussions, written informed consent was obtained following the principles of the Declaration of Helsinki (1996) [44]. Participants were provided with information and consent forms, available in both Norwegian and English, and any concerns or questions were addressed through interactive sessions [45]. The research adhered to ethical norms by utilizing audio recordings and note-taking during interviews. Participant data were anonymized with respondents (R) 1–31 (i.e., R1, R2., R31), coded as M for male and F for female, and stored in a secure location accessible only to authorized researchers to ensure confidentiality. The data analysis and presentation, including published works, were approached with meticulous care to safeguard participants’ identities.

## Findings

The demographic characteristics of the participants in this study reflect a diverse group of 31 first-generation immigrants, comprising 18 men and 13 women, aged between 27 and 66 years. All participants were born outside the host country and resided there for periods ranging from 2 to 16 years. The majority (*n* = 24) held permanent residency status, while a smaller group (*n* = 7) were temporary residents. In terms of educational attainment, 18 participants completed higher education, whereas 13 had secondary-level education. Employment status varied: 13 participants were employed full-time, 10 were employed part-time, four were unemployed, 3 were students, two were retired, and two were self-employed. It’s worth noting that access to health insurance was generally high, with 24 participants reporting full access, 5 indicating limited access, and 2 having no access at all. From the qualitative study of the respondents (*n* = 31), three key themes emerged: (1) cultural perceptions and health beliefs, (2) communication barriers and healthcare trust, and (3) navigating healthcare systems and the role of social networks.

### Cultural perceptions and health beliefs

Participants expressed that their health beliefs were strongly influenced by cultural and religious worldviews. Many viewed illnesses through a spiritual lens, attributing poor health to supernatural causes or spiritual imbalances rather than biomedical explanations.

“From my perspective, cultural and religious beliefs significantly influence my health. I have strong faith in the power of prayer or divine intervention, leading me to prioritize spiritual remedies or seek religious guidance as an initial response before consulting medical professionals.” (R30, F).

This belief in divine or spiritual causality often meant that prayer or consultation with religious leaders was the first step in addressing health concerns. Some participants described traditional healing as a vital and complementary part of their healthcare approach.

“Traditional healing practices have been passed down through generations. These practices often involve herbal remedies, spiritual rituals, or body-based therapies. For many of us, traditional healing practices complement or supplement Western medicine. I believe in Western medicine, but I try traditional medicine before going to the hospital.” (R1, M).

In addition to spiritual explanations, several participants mentioned cultural practices that emphasized traditional medicine as a trusted form of care. Remedies such as herbal infusions, roots, and rituals were common, particularly among those who had grown up with limited access to biomedical healthcare.

“In our culture, traditional medicine holds great significance. When faced with health issues, it is common for us to first seek traditional remedies and treatments from traditional healers or friends within our community.” (R13, M).

Although many preferred traditional medicines, others demonstrated a clear trust in Western medicine and evidence-based treatments. These participants tend to value formal healthcare services, especially when managing chronic or serious conditions.

“Some prefer conventional medical care and rely on Western medicine for their healthcare needs. We value evidence-based treatments and trust the expertise of healthcare professionals.” (R3, M).

Several participants stressed the value of integrating both systems to support holistic well-being—addressing physical and spiritual health together.

“I first seek traditional remedies before considering formal healthcare providers. The combination of traditional healing and Western medicine is beneficial for my body.” (R2, M).

Finally, participants highlighted specific traditional remedies they used for various health benefits, including immune support, digestion, and skin conditions. These included neem, moringa, ginger, kola, and rooibos tea—remedies they associated not only with cultural identity but also with effective health maintenance.

“Moringa and ginger have been lifesavers for me. They are packed with essential vitamins and minerals, and they really boost my immune system.” (R5, M).

Overall, participants expressed a dynamic interplay between cultural tradition and adaptation to new healthcare systems. For many, traditional medicine offered familiarity, cultural continuity, and holistic care, while others navigated both systems to meet their evolving health needs.

### Communication barriers and trust in healthcare

Language barriers, cultural misunderstandings, and differing health beliefs often hindered trust-building between patients and healthcare providers. Participants highlighted the importance of culturally competent healthcare professionals who communicate, respect diverse health beliefs, and engage in dialogue that bridges cultural gaps.

#### Language and interpretation challenges

Several participants emphasized how limited Norwegian proficiency hindered their ability to understand medical instructions or accurately express symptoms. Many stressed the need for qualified interpreters, noting that inadequate language support only amplifies existing communication barriers:

“Through personal encounters, I have witnessed firsthand how inadequate interpretation services can worsen the existing language barriers in healthcare. It is disheartening to see patients struggle to communicate their needs and concerns.” (R4, M).

Participants argued that interpreters are not just helpful but essential for ensuring accurate diagnosis and fostering mutual understanding. Some also urged healthcare providers to communicate in more accessible languages and to remain open to traditional perspectives:

“It is essential for healthcare professionals to communicate in a manner we can understand and to be open-minded and respectful of our traditional healing or medicine practices.” (R28, F).

#### Trust and perception of care

Trust in healthcare services was tied to perceived quality of care and the extent to which providers respected patients’ values and experiences. While some participants shared positive encounters, others felt dismissed or misunderstood:

“After many years in Norway, I have had both positive and negative experiences. Some providers have been excellent, while others rushed through appointments or didn’t take my concerns seriously.” (R30, F).

Negative experiences could diminish trust, especially when providers seemed hesitant or uncertain in their approach:

“Some doctors used to say they would *try* this treatment… Why do they try treatment? They must treat.” (R9, M).

However, a few respondents felt heard and respected, especially when healthcare providers acknowledged the role of traditional healing in their lives. This recognition fostered trust and satisfaction:

“Understanding and acknowledging the role traditional healing practices play in our lives and being willing to discuss their integration with Western medicine, can foster trust and collaboration.” (R12, M).

#### Culturally sensitive care

Effective communication and cultural sensitivity are fundamental to quality healthcare. Participants noted that when providers took the time to listen, explained clearly, and respected traditional practices, it strengthened the therapeutic relationship and improved healthcare outcomes.

### Navigating healthcare systems and the role of social networks

Respondents described the challenges of navigating a new and unfamiliar healthcare system, particularly when trying to balance the biomedical model in Norway with traditional healthcare practices from their countries of origin. This process involved reconciling differing cultural beliefs and health-seeking behaviors.

One participant explained:

“In my personal experience, navigating the healthcare system has been a significant challenge. The contrast between my home country’s healthcare practices and cultural beliefs and those in my host country adds complexity to my healthcare decisions, making it essential to find a balance between the two systems.” (R7, M).

Others emphasized the importance of integrating both systems:

“I believe in a holistic approach to health. While Western medicine is effective for acute illnesses, traditional remedies often address the root causes of chronic conditions.” (R14, M).

These accounts illustrate how navigating between two systems requires cultural sensitivity from healthcare providers and adaptability from patients, who often feel caught between contrasting worldviews.

In addition to the healthcare system itself, participants highlighted the pivotal role of family and religious institutions in health-related decisions. These networks provided emotional support, spiritual guidance, and practical assistance, shaping healthcare choices.

“In our culture, family plays a central role in health-related decisions. Their support and guidance are crucial during illness.” (R25, F).

“Religious institutions also play a role in health-related decisions. Many of us turn to our religious leaders for advice and seek comfort in our faith.” (R13, M).

These perspectives demonstrate the collective nature of decision-making and the centrality of spiritual and familial values in the healing process. They suggest that healthcare professionals should consider these dynamics when communicating with patients from similar cultural backgrounds.

A third theme centered on the importance of social networks in navigating and accessing healthcare. Friends, family, and community members often act as intermediaries and guides within the healthcare system.

“Our reliance on social networks is pivotal. We depend on advice and referrals to find healthcare providers who understand our cultural context.” (R1, M).

“My friends helped me schedule appointments, find interpreters, and even accompany me to the hospital.” (R16, F).

Such networks were described as essential for sharing practical information and reducing the isolation that many immigrants experience in accessing services. Those with limited support, by contrast, faced significant barriers.

“Having a supportive community or family unit is beneficial. They can help with transportation, interpretation services, and appointment scheduling.” (R25, F).

These findings highlight the structural importance of social connections in overcoming linguistic, logistical, and cultural obstacles to healthcare access.

Participants often experienced significant difficulties when navigating the Norwegian healthcare system, particularly in reconciling traditional and biomedical approaches. However, the presence of strong social networks—including family, friends, and religious institutions—played a crucial role in overcoming these challenges. These networks provided not only emotional and spiritual support but also practical assistance, underscoring the need for healthcare systems to recognize and engage with the broader social contexts of immigrant patients.

## Discussion

### Cultural perceptions and health beliefs

Our findings reveal how cultural and religious beliefs profoundly impact the healthcare-seeking behaviors and treatment preferences of West African immigrants residing in Norway, highlighting the diverse perceptions regarding health and illness. Understanding and respecting these cultural differences is crucial for providing effective and culturally sensitive healthcare services to this population [46]. Their beliefs diverge from the Western biomedical model and encompass a holistic approach that considers spiritual, emotional, and physical well-being [47]. Many traditional healing practices of West African immigrants focus on restoring balance and harmony within the individual, often utilizing methods such as herbal medicine, acupuncture, and energy healing. These are rooted in cultural traditions passed down through generations [48]. Ultimately, such beliefs are pivotal in shaping when, how, and from whom these individuals seek medical care [49, 50].

Moreover, beliefs about health and illness vary widely, with some respondents attributing illnesses to supernatural forces, environmental factors, or imbalances within the body [51]. Views on traditional [27] versus Western medicine differ across West African communities, influencing how immigrants engage with healthcare in Norway. Attitudes toward mental health and help-seeking also vary, impacting their perceptions of the healthcare system [51].

A prominent theme that emerged from this study is the integration of traditional healing practices with Western medicine. Many respondents preferred traditional remedies as the first course of action or as a supplement to Western medical interventions. This can be attributed to several factors. Traditional medicines are often perceived as a more holistic and natural treatment option, addressing physical symptoms as well as emotional and spiritual well-being [52]. Furthermore, traditional healers may be more accessible and affordable than Western medical professionals, particularly for individuals with limited financial resources [53]. Some respondents believe that traditional remedies have fewer side effects than Western medicine [54]. For example, individuals who believe that an illness is caused by spiritual imbalances may seek spiritual healing practices in addition to or instead of Western medical interventions. Cultural and religious beliefs are integral to this process and may delay an individual’s decision to seek Western medical care [55].

Understanding the cultural and religious beliefs of West African immigrants is crucial for healthcare providers in Norway to build trust and ensure comprehensive, patient-centered care [56, 57]. The findings of this study emphasize that this extends beyond mere sensitivity—it is a fundamental requirement for effective care. Instead of a general call for “cultural competence training,” this study highlights specific areas for professional development. Effective training must equip providers to navigate conversations about holistic health models, the role of spirituality in healing, and the integration of traditional remedies with biomedical treatments. An essential aspect of this professional and cultural awareness requires healthcare providers to consider both their own values and attitudes and those of their patients, complementing their existing knowledge and skills [58, 59].

To facilitate successful healthcare integration—a process involving mutual adaptation where the host society accommodates diversity [60]—this study puts forward specific, actionable recommendations. A primary recommendation is to foster formal collaboration between Norwegian healthcare providers and traditional healers. This would create an integrated care model that respects and utilizes the strengths of both Western and traditional medicine [61]. Such a model should be supported by educating patients on the benefits and limitations of both approaches, empowering them to make informed decisions while upholding evidence-based practices.

By adopting such a specific and culturally responsive approach [59], healthcare providers can improve health outcomes for West African immigrants and promote more equitable access to healthcare services.

### Communication barriers and trust in healthcare

The integration of West African immigrants into the Norwegian healthcare system is significantly impacted by cultural barriers and language disparities. These challenges often hinder effective communication and trust-building between patients and healthcare providers, as noted by several participants. Many respondents highlighted the difficulties in understanding medical instructions and their inability to explain symptoms or ask questions due to language limitations. While cultural barriers may include differences in healthcare practices and beliefs, language disparities further complicate effective communication between patients and providers [58].

Language and communication barriers present substantial obstacles to accessing healthcare services [62, 63]. Al Shamsi et al. (2020) discussed how language barriers hindered effective communication between and satisfaction among medical professionals and patients, compromised high-quality care, and jeopardized patient safety. Limited language proficiency has been associated with serious consequences for healthcare quality, including misdiagnoses, inappropriate treatments, and increased adverse events [64, 65]. For instance, a study analyzing incident reports from six U.S. hospitals found that limited English proficient (LEP) patients experienced physical harm at a higher rate (49.1%) compared to English-speaking patients (29.5%) [64, 66].

When patients struggle to convey their symptoms or understand medical advice due to language barriers, healthcare providers may miss crucial information, leading to mistrust, misdiagnosis, inappropriate interventions, and negative patient outcomes [63]. Such misunderstandings can erode trust between patients and providers, compromising the quality and acceptance of treatment [46, 58]. For example, some West African cultures may prioritize spiritual or traditional healing practices, while the Western biomedical model emphasizes physical and biological factors. This divergence in health perspectives can create tension. This is often aggravated due to poor communication, resulting in mistrust and reluctance to seek conventional medical care [58].

The consequences of these communication failures are significant, ranging from compromised patient safety to a deep erosion of trust [63, 64]. These communication failures are not merely logistical problems; for many patients, they are perceived as signs of disrespect or bias, which profoundly erodes the trust necessary for a good therapeutic relationship. To counteract this, providers need to implement specific, practical communication strategies. The most critical step is the consistent use of qualified professional interpreters rather than relying on family members, who may filter information or lack precise medical terminology [62]. Beyond formal interpretation, providers must employ techniques such as using clear, jargon-free language and practicing active listening to confirm patient understanding and validate their concerns [67]. Furthermore, actively involving patients in shared decision-making is fundamental to shifting the dynamic from misunderstanding to collaboration [68]. By focusing on these concrete communicative actions, the healthcare system can directly address the trust deficit, mitigate safety risks, and ensure more equitable care for West African immigrants in Norway [46].

### Navigating healthcare systems and the role of social networks

As previously established, the Norwegian healthcare system presents significant navigational challenges for many West African immigrants due to its complexity and cultural dissonance [69, 70]. Our findings reveal that in response to these barriers, individuals rely heavily on informal social networks—including family, friends, and religious leaders—to access care. These networks provide critical support, from practical assistance with scheduling appointments and translation to offering advice and reassurance rooted in a shared cultural understanding [30, 40]. The level of reliance on these networks appears greatest among newly arrived immigrants, who have less familiarity with the Norwegian biomedical system [71, 72].

The crucial role of these informal support systems points to a clear, actionable strategy for the healthcare system: to formally integrate this community-based expertise into the care model. Instead of leaving patients to rely on ad-hoc help, a more effective approach would be to fund and implement programs with ‘patient navigators’ or ‘cultural liaisons’ (helseloser) from West African communities. These liaisons could officially bridge the linguistic and cultural gap between patients and providers, assist in navigating administrative processes, and ensure that health information is understood in a culturally relevant context.

Furthermore, developing the necessary patient education initiatives requires active collaboration with trusted community-based organizations and faith-based groups [72, 73]. By co-designing resources with these partners, the healthcare system can ensure that information is not only linguistically and culturally appropriate but also distributed through established and trusted channels. In tandem with more flexible care models like telehealth and home visits, this community-integrated approach represents a fundamental shift from passive awareness to active partnership, ensuring more equitable and navigable healthcare for West African populations.

This discussion argues that improving healthcare for West African immigrants in Norway requires a fundamental shift from passive cultural awareness to an active, structural partnership model. The challenges identified—from differing health beliefs and communication breakdowns to difficulties navigating the system—are not isolated incidents. Instead, they are often symptoms of deeper structural barriers, which can include systemic biases and perceived discrimination that leave patients feeling dismissed or disrespected.

Therefore, the solutions must also be structural. Our findings indicate that progress depends on a multi-pronged strategy. The foundation is to formally integrate community expertise into the healthcare system by actively collaborating with traditional healers and employing patient navigators who can bridge cultural and systemic divides. This must be supported by implementing specific communication protocols, including the mandated use of professional interpreters and training in patient-centered dialogue to build trust actively. Finally, adapting care delivery through more flexible models can address practical barriers.

By embedding these concrete, community-partnered solutions into the Norwegian healthcare system, it is possible to move beyond a ‘one-size-fits-all’ approach. These are not simply ‘cultural accommodations’; they are essential structural changes required to dismantle barriers, counteract discrimination, and lay the foundation for truly equitable healthcare for all.

## Strengths and limitations of the study

This study offers several noteworthy strengths. First, it addresses a critical gap in the literature by focusing specifically on West African immigrants in Norway—an understudied group in migration health research. By centering their cultural perspectives and health beliefs, the study contributes to a more nuanced and inclusive understanding of healthcare access and use among diverse migrant populations. Second, the relatively large sample size of 31 participants is a definite strength. It allows for the generation of rich and diverse insights, especially considering the heterogeneity of immigrant experiences. The diversity of the sample in terms of country of origin, gender, language, and socioeconomic background further enhances the transferability of the results. Additionally, the researcher’s insider–outsider positionality and use of culturally responsive methods helped foster trust and openness, strengthening the authenticity and credibility of the data.

Limitations of the study include the non-random, purposive sampling method and the voluntary nature of participation, which may introduce self-selection bias. Although efforts were made to include diverse perspectives, the sample may not fully represent all West African immigrants in Norway. Nevertheless, the depth of qualitative insight provides valuable contributions to understanding immigrant healthcare experiences.

### From awareness to action: a blueprint for equitable healthcare for West African immigrants

With an increasing number of immigrants from African countries residing in Norway, West Africans represent a significant and growing segment of the population that requires more equitable and culturally responsive healthcare services. To meet their needs effectively, the Norwegian healthcare system must adopt a comprehensive strategy that combines structural political reform with community-based, evidence-informed practices. This population often faces intersecting barriers—language, discrimination, and difficulties navigating a complex and unfamiliar health bureaucracy. Addressing these challenges demands not only political will but also sustained, culturally informed interventions.

A fundamental shift should begin by embedding cultural competence at the core of healthcare education and clinical practice. Rather than treating it as an optional module, cultural competence should become a requirement for licensure and re-certification for all licensed health professionals. Training must be developed in collaboration with leaders from West African communities to reflect culturally specific practices, such as collective family involvement in healthcare decisions, the influential role of faith and religious leaders, and the standard integration of traditional healing with biomedical treatment. Understanding diverse expressions of pain and psychological distress—as well as how gender roles influence health-seeking behavior—is essential to preventing misdiagnosis and improving care quality.

Equally important is reforming language access and communication infrastructure. Every patient should be guaranteed access to a professional, in-person medical interpreter. It is time to move away from inconsistent telephone services or the use of family members, which pose serious risks to clinical accuracy, patient confidentiality, and safety. A centralized, publicly funded interpreter corps—with training in medical terminology, confidentiality, and ethics—is critical. Health portals, appointment letters, and informational materials must be co-designed with immigrant communities and made available in relevant West African languages, including French and Pidgin English, where appropriate.

To combat systemic inequalities, it is crucial to acknowledge that perceived discrimination is not merely a subjective experience—it has measurable health consequences. Research on “weathering” has shown that chronic exposure to discrimination and structural stress contributes to higher rates of hypertension, diabetes, and other chronic conditions. Healthcare institutions must be required to implement explicit anti-discrimination policies and collect anonymized patient-reported experience data disaggregated by ethnicity. These data should be actively used to identify and eliminate disparities in treatment and outcomes. A transparent, independent reporting mechanism for discrimination—housed within the Equality and Anti-Discrimination Ombud but supported by a dedicated health division—could ensure accountability and enable timely intervention.

Another essential step is the formal integration of health navigators or cultural mediators into the healthcare system. Ideally recruited from the communities they serve, these individuals should undergo formal training in health literacy, confidentiality, and intercultural communication. In partnership with religious institutions, community centers, and cultural organizations, they can support patients in understanding treatment plans, scheduling appointments, and accessing preventive services or social supports such as housing or employment. Ethical frameworks for these roles should be developed in alignment with national health laws and in collaboration with patient advocacy organizations to ensure adherence to professional standards and maintain trust.

Alongside these systemic reforms, existing grassroots models offer valuable lessons. The Health Centre for Undocumented Migrants in Oslo, operated by the Red Cross and other volunteer actors, provides free healthcare without requiring identification [74]. While most West African immigrants in Norway have legal residency, this low-threshold model, characterized by accessibility, simplicity, and a welcoming environment, demonstrates how trust can be built and care extended to marginalized groups. Similar community-based clinics can be adapted to serve immigrant populations better.

In Sweden, the national Hälsa i Sverige program for asylum seekers and newly arrived immigrants has proven the scalability of training interventions [75]. Using a “train-the-trainer” model [76, 77], healthcare workers receive education on migration-related stress and culturally sensitive care, which they then deliver to their local colleagues. This model institutionalizes ongoing professional development, avoiding the inefficiency of one-off cultural competence courses.

In Norway, cultural and voluntary organizations already serve as informal bridges to the healthcare system. Recognizing and funding these groups as official partners would strengthen their capacity and ensure that their community insights directly inform healthcare delivery. A model similar to Norway’s “competence brokers,” which connect businesses to research and innovation, could be adapted to formally link immigrant communities (including respected traditional healers) with public health institutions, fostering trust and system relevance.

By implementing such reforms and drawing on grassroots innovation, Norway can move toward a healthcare system that more effectively serves its diverse population. For West African immigrants, this transformation would mean not just improved access, but respectful, equitable, and culturally attuned care that promotes both health and dignity.

## Conclusion

This study explored the factors that influence how individuals from West Africa engage with the Norwegian healthcare system. Utilizing a qualitative research design, the study captured rich, in-depth data reflecting the participants lived experiences and cultural contexts. The findings highlight the importance of culturally sensitive healthcare practices and emphasize the need for healthcare professionals to consider and respect the diverse perspectives that immigrant patients may bring. This study contributes to the growing field of culturally competent care by emphasizing culturally specific health beliefs and practices—often overlooked in mainstream healthcare research. It provides valuable insights into an under-researched population and offers important implications for healthcare professionals and policymakers aiming to enhance the accessibility and responsiveness of healthcare services in multicultural settings. Moreover, the results underscore the importance of fostering open dialogue and mutual understanding between patients and healthcare providers to bridge potential gaps in healthcare expectations and practices. In doing so, the study meaningfully contributes to the ongoing discourse on equitable and inclusive healthcare delivery. It reinforces the significance of embracing diversity within healthcare contexts, especially in a multicultural society like Norway. Continued research is essential to further support inclusive practices and ensure that all individuals, regardless of cultural background, receive respectful and effective care.

## Supplementary Information

Supplementary Material 1.

## Data Availability

The datasets produced and examined in this study are not publicly accessible because of privacy and confidentiality agreements with participants. However, they can be obtained from the corresponding author upon reasonable request, provided ethical approvals are in place.
