# Supplementary material for: Perceptions related to health, illness, and provision of healthcare among West African migrants residing in Norway: a qualitative study
Source: BMC Health Serv Res. 2025 Oct 9;25:1338. doi: 10.1186/s12913-025-13329-w (PMC12512893; doi:10.1186/s12913-025-13329-w)
Supplement: Supplementary file 1 — Supplementary Material 1. [file 12913_2025_13329_MOESM1_ESM.docx]

**Supplementary file 1 – Interview Guide**

This guide is designed to be flexible, allowing for follow-up questions or deeper exploration of responses as necessary, ensuring we capture the full depth of participants' experiences and perspectives.

**Topic: Perceptions related to health, illness, and provision of healthcare among West African migrants residing in Norway: A qualitative study**

The questions that will guide the interviews are:

How old are you?

In what country were you born?

What is you mother tongue?

Do you speak other languages?

When did you start learning Norwegian? Did you know it before you came to Norway?

Do you think the knowledge of Norwegian is important or not?

What language do you speak at home?

How long have you been in Norway? The years of stay in Norway?

Do you have relatives here in Norway?

Do you have or own a house?

How did you obtain your housing?

Was it difficult or not to obtain housing here?

Do you like your current living situation?

What kind of educational background do you have?

How satisfied are you?

Are you studying now? Do you have kids? Are your kids going to school?

Are you currently employed?

What is your occupation?

What type of job? Self-employed? How many hours a week?

How do you thing about access to healthcare?

How did you contact the healthcare services? telephone, webpage, e-consultation, meeting up)

Why did you you contact the healthcare services?

How did you experience the meeting with the healthcare services?

Did they understand your need?

How are you experience concerning access to healthcare? Can you tell me about any such experiences?

What kind of obstacles have you experienced in relating to access to healthcare?

Do you have any good experiences with the healthcare services?

Do you trust that the healthcare services?

How has your health been since you came to Norway?

Do you treat youselv? How do you treat yourselv?

How do you perceived your symptoms and health conditions

When and how do you see health caring?

What is your expectations of care?

What is your preferances regarding traitements?

What traditional health caring and medicines experiences and beliefs do you have or bring with you to Norway?

**Norwegian healthcare provider**

What do you know about the use of traditional health caring among west African immigrants in Norway?

What experiences do you have in health caring for patients from ethnically diverse populations within the Norwegian health care system?

What do you do to improve the health caring of immigrants in Norway?

Are there differences in health care for patients from other countries?

**Closing Remarks**

Is there anything else you would like to share that we haven’t discussed?

**Thank You Statement**

- Thank you so much for sharing your thoughts and experiences. Your input is very valuable and will contribute significantly to this research. If you have any questions or would like to follow up later, please feel free to contact me.
